# Pharmacokinetic Modeling of Targeted Ultrasound Contrast Agents for Quantitative Assessment of Anti-Angiogenic Therapy: a Longitudinal Case-Control Study in Colon Cancer

**DOI:** 10.1007/s11307-018-1274-z

**Published:** 2018-09-17

**Authors:** Simona Turco, Ahmed El Kaffas, Jianhua Zhou, Amelie M. Lutz, Hessel Wijkstra, Jürgen K. Willmann, Massimo Mischi

**Affiliations:** 1 Department of Electrical Engineering, Eindhoven University of Technology, Groene Loper 19, 5612 AZ Eindhoven, The Netherlands; 2 Department of Radiology, Stanford Medicine, Stanford, CA 94305 USA; 3 Department of Ultrasound, State Key Laboratory of Oncology in South China, Collaborative Innovation Center for Cancer Medicine, Sun Yat-Sen University Cancer Center, Guangzhou, China; 4 Department of Urology, Academic Medical Center, 1105 AZ Amsterdam, The Netherlands

**Keywords:** Ultrasound molecular imaging, Antiangiogenic therapy, Cancer therapy monitoring, Colorectal cancer, Pharmacokinetic modeling

## Abstract

**Purpose:**

To evaluate quantitative and semi-quantitative ultrasound molecular imaging (USMI) for antiangiogenic therapy monitoring in human colon cancer xenografts in mice.

**Procedures:**

Colon cancer was established in 17 mice by injection of LS174T (*N*_r_ = 9) or CT26 (*N*_n_ = 8) cancer cells to simulate clinical responders and non-responders, respectively. Antiangiogenic treatment (bevacizumab; *N*_rt_ = *N*_nt_ = 5) or control treatment (saline; *N*_rc_ = 4, *N*_nc_ = 3) was administered at days 0, 3, and 7. Three-dimensional USMI was performed by injection at days 0, 1, 3, 7, and 10 of microbubbles targeted to the vascular endothelial growth factor receptor 2 (VEGFR2). Microbubble binding rate (*k*_b_), estimated by first-pass binding model fitting, and semi-quantitative parameters late enhancement (LE) and differential targeted enhancement (dTE) were compared at each day to evaluate their ability to assess and predict the response to therapy. Correlation analysis with the *ex-vivo* immunohistological quantification of VEGFR2 expression and the percentage blood vessel area was also performed.

**Results:**

Significant changes in the USMI parameters during treatment were observed only in the responders treated with bevacizumab (*p*-value < 0.05). Prediction of the response to therapy as early as 1 day after treatment was achieved by the quantitative parameter *k*_b_ (*p*-value < 0.01), earlier than possible by tumor volume quantification. USMI parameters could significantly distinguish between clinical responders and non-responders (*p*-value << 0.01) and correlated well with the *ex-vivo* quantification of VEGFR2 expression and the percentage blood vessels area (*p*-value << 0.01).

**Conclusion:**

USMI (semi)quantitative parameters provide earlier assessment of the response to therapy compared to tumor volume, permit early prediction of non-responders, and correlate well with *ex-vivo* angiogenesis biomarkers.

## Introduction

Cancer is one of the leading causes of death worldwide and represents a major health challenge, accounting for 8.8 million deaths in 2015—about 16 % global mortality [1]. Among all cancers, colorectal cancer is particularly relevant, representing the third most prevalent and second most lethal cancer in man and women combined in the USA [2].

Based on the established link between cancer growth and the process of angiogenesis [3, 4], *i.e.*, the formation of a vascular network supporting tumor development, novel therapeutic strategies aim at blocking or disrupting specific angiogenic pathways [5–7]. Due to its ubiquitous overexpression, the vascular endothelial growth factor (VEGF) is the dominant target of antiangiogenic drugs. Currently, several VEGF inhibitors are approved for first and second lines of treatment of different types of cancers in the USA and Europe. In colon cancer, VEGF pathways represent one of the main targets for treatment of metastatic disease [6–9].

Early evaluation of the therapeutic response is crucial to identify potential non-responders, allowing for better therapy tailoring and patient management. Current therapy assessment criteria based on survival time and tumor dimension are not suitable for evaluation of early response, especially in the case of novel antiangiogenic therapies, which act by interfering with angiogenic processes and may not lead to any change in tumor size [10]. Biomarkers for early assessment of antiangiogenic therapies are thus needed to improve therapeutic decision-making [11].

Activation of intratumoral angiogenesis through the VEGF pathway involves the phosphorylation of VEGF receptors overexpressed on tumor vasculature [12, 13]. Among these, the VEGF receptor 2 (VEGFR2) has been established as the main receptor in human malignancies [12], showing up to five times higher expression in tumor compared to normal vasculature [13]. Under the hypothesis that the overexpression of VEGF may mediate the upregulation of VEGFR2, imaging probes targeting VEGFR2 may represent a promising option for assessment of cancer therapies inhibiting the VEGF/VEGFR2 pathways [12–15].

Portability, low cost, availability, and absence of ionizing radiations make ultrasound imaging a promising option for antiangiogenic therapy monitoring, whereby several repeated exams are needed. Ultrasound molecular imaging (USMI) of angiogenesis has become possible by the introduction of novel targeted ultrasound contrast agents (tUCAs) [16]. These are composed of microbubbles which can flow through the vasculature and attach to the vessel walls where the target molecule is overexpressed, thus causing selective enhancement in areas of active angiogenesis. In this context, the clinical grade tUCA BR55 targeting VEGFR2 was recently developed and tested for human use [17–19].

Evaluation of the degree of microbubble binding has shown to be a promising biomarker of angiogenesis [14, 20–22]. Semi-quantitative assessment is typically achieved by evaluating the late enhancement, several minutes after injection. Especially in preclinical studies, a more quantitative evaluation has been achieved by application of a high-pressure ultrasound burst to calculate the differential targeted enhancement (dTE), *i.e.*, the difference in the image intensity before and after microbubble destruction [14, 16, 21, 23–26]. Semi-quantitative USMI, however, is user- and machine-dependent, it requires lengthy procedures (~ 5–10 min in animals), and when a destructive burst is applied, it raises concerns for damages to the endothelial tissue [27].

Quantitative assessment may overcome these limitations. Several mathematical models have been proposed to describe the tUCA kinetics, which are based either on purely empirical models [28, 29] or on the combination of physiological and empirical models [30] or on pharmacokinetic modeling [31, 32]. Fitting these models to time-intensity curves (TICs) measured with USMI enables the estimation of quantitative parameters related to cancer angiogenesis. In this context, the first-pass binding (FPB) model enables characterization of microbubble binding by the estimation of the binding rate (*k*_b_) [32, 33]. By focusing only on the first pass of the contrast bolus, this method is not affected by potential inaccuracies due to contrast recirculation and enables reducing the required USMI acquisition time to about 1 min.

The purpose of this study was to evaluate semi-quantitative and quantitative USMI for assessment of the early response to antiangiogenic treatment on colon cancer-bearing mice monitored during therapy.

## Material and Methods

### Tumor Model

**Fig. 1.**
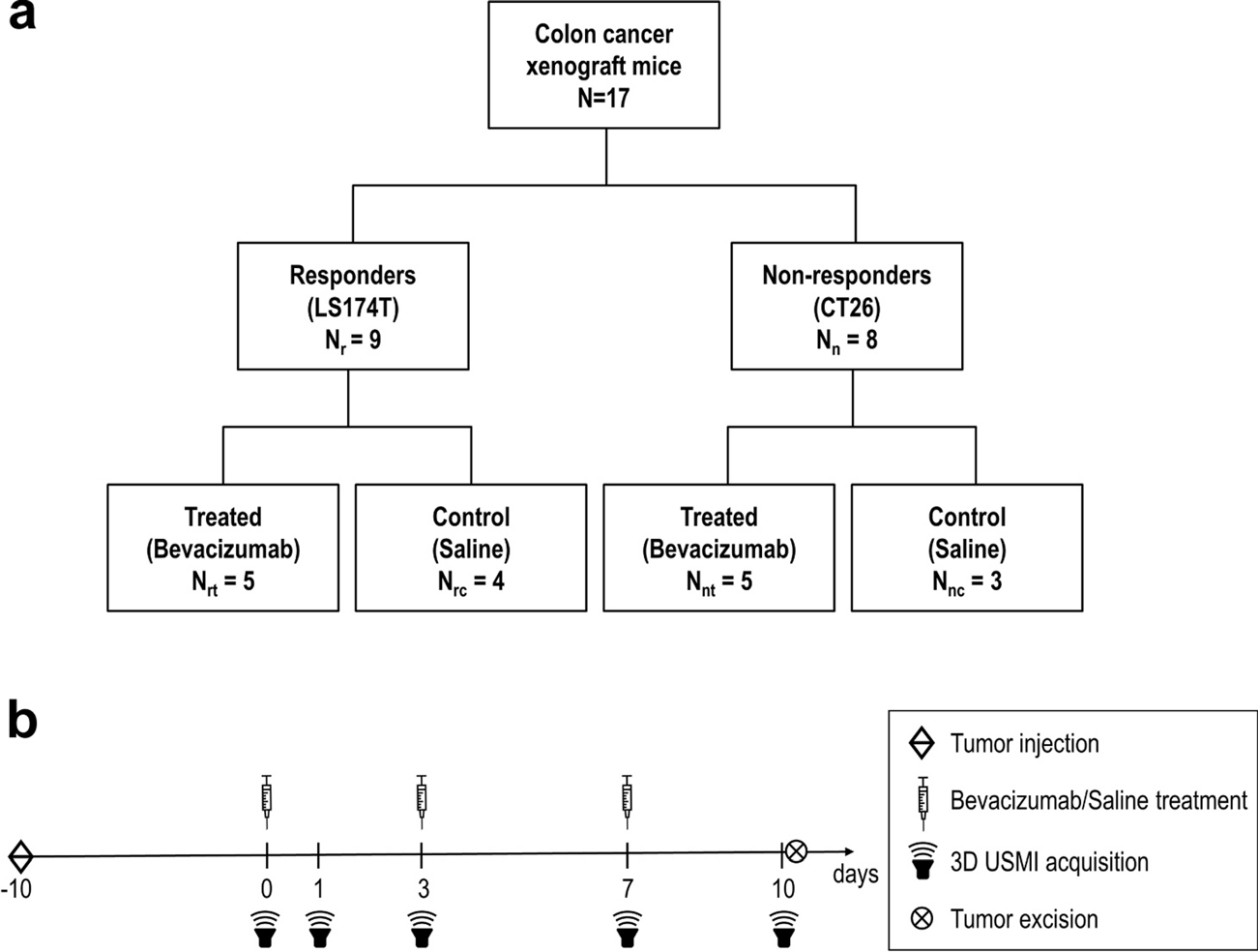
**a** Flow chart summarizing the mice dataset and the treatment/control randomization. **b** Timeline of the animal experiments: after letting tumor grow for 10 days, 3D USMI is performed by injection of BR55 at baseline (day 0) and repeated at days 1, 3, 7, and 10; treatment is performed by injection of either bevacizumab (treated groups) or saline (control groups) at day 0 and repeated at days 3 and 7; 10 days after treatment initiation, the mice are sacrificed and the tumor are excised for immunohistological quantification.

All animal experiments were approved by the Institutional Administrative Panel on Laboratory Animal Care at Stanford University. Colon cancer xenografts were established in 6–8-week-old female athymic nude mice (*N* = 17) obtained from Charles River Laboratories (Wilmington, MA). Clinical responders (*N*_r_ = 9) and non-responders (*N*_n_ = 8) were simulated by subcutaneous injection of, respectively, the human colon cancer line LS174T, which has been shown to be sensitive to VEGF-targeted treatment [6], and the murine colon cancer cell line CT26, which has shown resistance to VEGF-targeted treatment [34]. Both cell lines were obtained from ATCC (Manassas, VA). After cell injection, tumors were allowed to grow for 10 days to a maximum diameter of 6–13 mm. The mice were then randomized into a treatment group, including five responders and five non-responder mice (*N*_rt_ = *N*_nt_ = 5), and a control group, including four responders (*N*_rc_ = 4) and three non-responders (*N*_nc_ = 3). A flow chart summarizing the mice dataset is shown in Fig. 1a. On days 0, 3, and 7, the treatment group received a 10 mg/kg intravenous injection of bevacizumab (Avastin^®^; Genentech, South San Francisco, CA), while the control group was injected with sterile saline (Fig. 1b).

### Three-Dimensional USMI

Three-dimensional USMI was performed at days 0, 1, 3, 7, and 10, by a 100 μl injection of BR55 (Bracco Suisse, Geneva, Switzerland) in the mouse tail vein through a 27-G needle catheter (Vevo MicroMarker; VisualSonics, Toronto, Canada) at a constant injection rate of 20 μl/s using an infusion pump (Kent Scientific, Torrington, CT). The ability of BR55 to recognize both human and murine VEGFR2 has been previously shown [17]. Contrast imaging in power modulation mode was performed with an EPIQ7 clinical ultrasound system (Philips Healthcare, Andover, MA) equipped with an X6-1 matrix array probe working at 3.2 MHz (frame rate = 1 Hz, dynamic range = 52 dB, focal depth = 5 cm). The mechanical index (MI) was initially kept constant at 0.09 to allow microbubble circulation and binding to target sites. Three-dimensional frames were acquired at a frame rate of 1 Hz for a total duration of about 5 min. After about 4 min from the start of the acquisition (*t* = *t*_flash_), a destructive burst was applied by increasing the MI to 0.72 for 2 s. The MI was then switched back to 0.09 to image microbubble replenishment.

### Pharmacokinetic Modeling of the Binding Kinetics of Targeted Contrast Agents

The total concentration of a targeted ultrasound contrast agent in a pixel of tissue, *C*(*t*), can be expressed as the weighted sum of the concentration of free microbubbles, *C*_f_(*t*), and bound microbubbles, *C*_b_(*t*), as

1 $$ {C}_{\mathrm{t}}(t)={v}_{\mathrm{f}}{C}_{\mathrm{f}}(t)+{v}_{\mathrm{b}}{C}_{\mathrm{b}}(t) $$

where *v*_f_ and *v*_b_ are the fractional volumes of free and bound microbubbles, respectively.

In general, the free transport of microbubbles in a blood vessel can be described as a convective dispersion process by which the linear translation of the microbubbles dragged by the carrier fluid (*i.e.*, blood) is superimposed to the microbubble dispersion by Brownian motion. The modified local density random walk (mLDRW) model provides a local characterization of the dispersion process by which the concentration of free microbubbles after a fast bolus injection can be described as [35]

2 $$ {C}_{\mathrm{f}}(t)=\alpha \sqrt{\frac{\kappa }{2\pi \left(t-{t}_0\right)}}{e}^{-\frac{\kappa {\left(t-{t}_0-\mu \right)}^2}{2\left(t-{t}_0\right)}} $$

where *α* is the integral of *C*_f_(*t*), *μ* is the mean transit time of microbubbles between injection and detection sites, *t*_0_ is the theoretical injection time, and *κ* is the local dispersion parameter given by the ratio between the squared convection velocity (*v*^2^) and the dispersion coefficient (*D*).

If negligible microbubble unbinding can be assumed in the first pass of the injected bolus, the kinetics of bound microbubbles can be described by a well-mixed accumulating compartment as

3 $$ {v}_{\mathrm{b}}\frac{\partial {C}_{\mathrm{b}}(t)}{\partial t}={k}_{\mathrm{b}}{C}_{\mathrm{f}}(t) $$

where *k*_b_ is the binding rate constant, which is adopted to quantify microbubble binding. With initial conditions *C*_b_(*t* = 0) = *C*_f_(*t* = 0) = 0, Eq. (3) can be solved as

4 $$ {v}_{\mathrm{b}}{C}_{\mathrm{b}}(t)={k}_{\mathrm{b}}\varTheta (t)\ast {C}_{\mathrm{f}}(t) $$

where the symbol * represents the convolution integral and Θ(*t*) is the Heaviside unit step function. If the adiabatic approximation is made [12], by which the binding kinetics is assumed to be much slower than the free transport kinetics (*i.e.*, *κ* >> *k*_b_), *C*_f_(*t*) can be modeled as in Eq. (2), and it can be substituted in Eqs. (4) and (1). This leads to the FPB model described by [32]

5 $$ {\displaystyle \begin{array}{l}{C}_{\mathrm{t}}(t)={v}_{\mathrm{f}}{C}_{\mathrm{f}}(t)+{k}_{\mathrm{b}}\ast {C}_{\mathrm{f}}(t)=\\ {}\kern2.12em ={v}_{\mathrm{f}}\alpha \sqrt{\frac{\kappa }{2\pi }}{\left(t-{t}_0\right)}^{-1/2}{e}^{-\frac{\kappa {\left(t-{t}_0-\mu \right)}^2}{2\left(t-{t}_0\right)}}+{k}_{\mathrm{b}}\alpha \sqrt{\frac{\kappa }{2\pi }}\underset{0}{\overset{\tau }{\int }}{\left(\tau -{t}_0\right)}^{-1/2}{e}^{-\frac{\kappa {\left(t-{t}_0-\mu \right)}^2}{2\left(t-{t}_0\right)}} d\tau .\end{array}} $$

### US Quantification

Tumor volumes were manually segmented on each 3D USMI dataset in random order by a reader blinded to the treatment randomization, using custom software developed in MeVisLab (MeVis Medical Solutions AG, Bremen, Germany). Tumor volume was quantified by measuring the greatest longitudinal (*L*), transverse (*W*), and anteroposterior (*H*) dimensions of tumors on 3D B-mode US prior to contrast injection and by using the formula *V* = *π* / 6 *L* · *W* · *H*. For each 3D USMI dataset, a linearized TIC was obtained at each pixel of each 2D imaging plane. Semi-quantitative analysis was performed by calculation of the late enhancement (LE), evaluated as the gray level (g.l.) at the empirically chosen time point *t* = *t*_flash_ − 40 s, and the dTE, calculated as the difference between the average g.l. for the empirically chosen time intervals *t* > *t*_flash_ + 30 s (after the destructive US burst) and *t*_flash_ − 130 s < *t* < *t*_flash_ − 30 s (before the destructive US burst). Quantitative analysis was performed by fitting the first minute of each voxel TIC to FPB model in Eq. (5) to estimate the microbubble binding rate (*k*_b_, min^−1^). Fitting was performed by non-linear least-squares curve fitting with the trust region reflective method [32, 36]. Since the estimated parameters are not Gaussian distributed in each 2D plane, the median value of each USMI parameter was first calculated for each 2D imaging plane. For statistical analysis, a mean value for the whole 3D volume of each USMI dataset was then obtained by averaging over all the 2D planes. All the analysis was implemented in MatLab^®^ (MathWorks, Natick, MA) running on a desktop computer.

### Immunohistological Quantification

At day 10, mice were sacrificed for tumor *ex-vivo* analysis. After 24-h fixation in a solution of 4 % paraformaldehyde and phosphate-buffered saline, followed by 3-day fixation in 30 % sucrose and phosphate-buffered saline solution (Sigma-Aldrich, St Louis, MO), tumors were sectioned into 10-mm slices for immunofluorescence staining. Rabbit antimouse VEGFR2 antibody (Cell Signaling, Danvers, MA) and rat antimouse CD31 antibody (eBioscience, San Jose, CA) were used to quantity VEGFR2 expression and the percentage blood vessel volume, respectively. Fluorescent microscopy was performed with an LSM510 meta-confocal microscope (Zeiss, Maple Grove, MN) attached to a digital camera (AxioCam MRc, Bernried, Germany) using a × 20 objective. On each histological slice, five fields of view (FOVs) of 0.19 mm^2^ were randomly selected and the VEGFR2 expression and the percentage blood vessel area per FOV were quantified with ImageJ software (National Institutes of Health, Bethesda, MD) as the average value in the five FOVs.

### Statistical Analysis

For each combination of tumor model (responder/non-responder) and treatment (treated/control), the USMI parameter values before and after treatment were compared by performing the Kruskal-Wallis test. Post hoc analysis was performed by the Wilcoxon-Mann-Whitney two-sample test comparing parameter values at days 1, 3, 7, and 10 with day 0, adjusting the *p*-value for the number of multiple comparison, according to the Benjamini-Hochberg (BH) procedure [37]. The ability of (semi)quantitative USMI of distinguishing between clinical responders and non-responders was evaluated on the treated groups by performing the BH-adjusted Wilcoxon-Mann-Whitney two-sample test on the fold changes in the parameter values at each day after day 0 (baseline). The Pearson linear correlation and the Spearman rank correlation coefficients were calculated to assess (i) the correlation among the USMI parameters, including the values obtained at all days, and (ii) the correlation of the *in-vivo* USMI parameters with the *ex-vivo* assessment of VEGFR2 expression levels and the percentage blood vessel area, pooling the USMI parameter values obtained at days 7 and 10 (two last acquisitions prior to tumor excision) to reduce the variance resulting from the small sample size. All statistical analysis was performed in MatLab.

## Results

### Early Assessment of Therapeutic Response

**Fig. 2.**
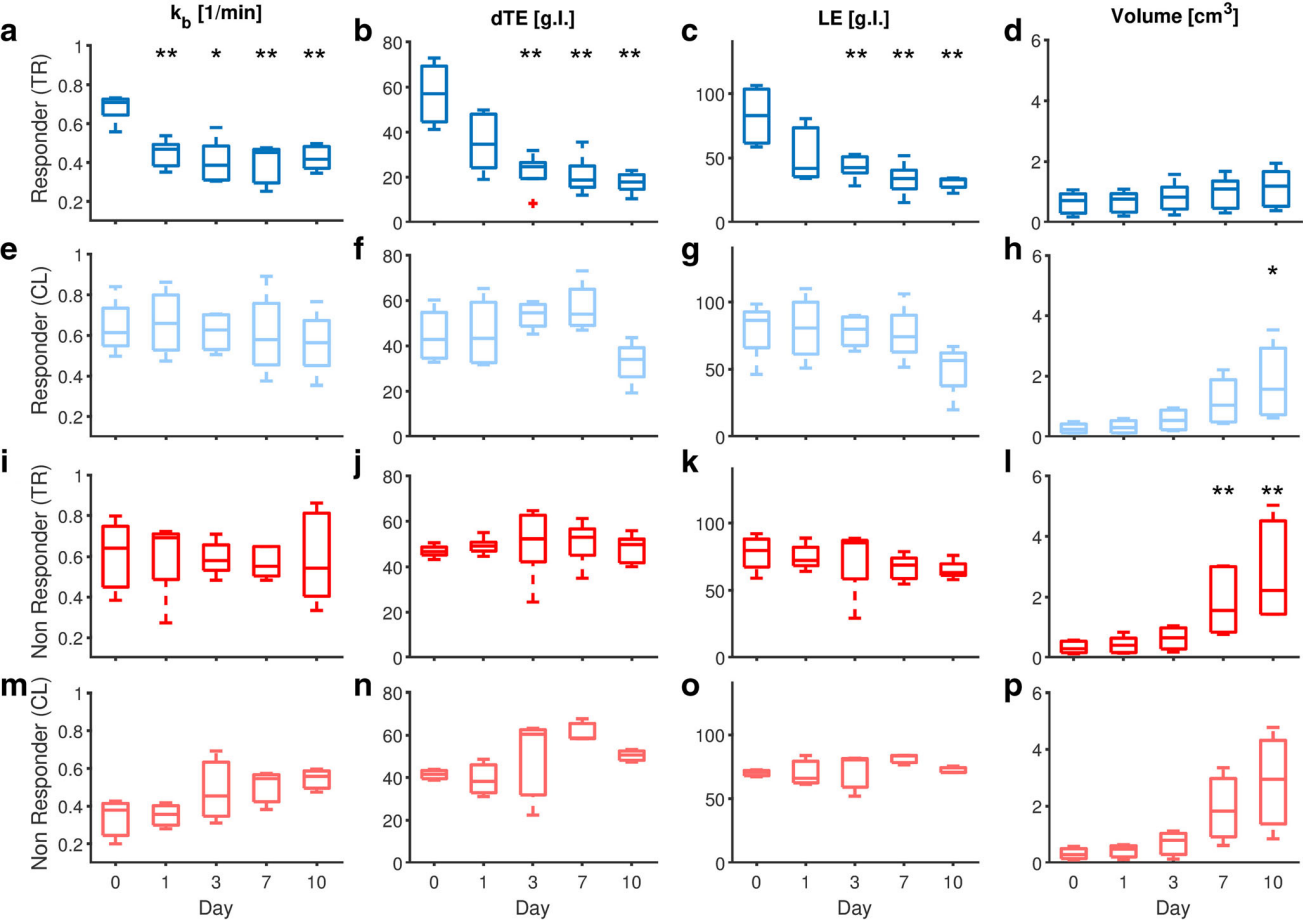
**a**–**p** Box plots comparing USMI parameter values and tumor volume before and after treatment. Significant differences with respect to day 0, as tested by ANOVA analysis with the Tukey HSD post hoc test, are indicated by asterisks (*p-value < 0.05, **p-value < 0.01).

The changes in USMI parameters and tumor volume during treatment are shown in Fig. 2. In the responders treated with bevacizumab, significant differences in *K*_b_ (*p*-value < 0.01) were observed after 1 day post treatment (Fig. 2a) and those in dTE (*p*-value < 0.01) and LE (*p*-value < 0.01) after 3 days post treatment (Fig. 2b, c). No significant changes during treatment were instead observed for the responders in the control group (*K*_b_: *p*-value > 0.34; dTE: *p*-value > 0.34; LE: *p*-value > 0.2) (Fig. 2e–g) and for the non-responders in both the treated (*K*_b_: *p*-value > 0.69; dTE: *p*-value > 0.69; LE: *p*-value > 0.15) and control (*K*_b_: *p*-value > 0.4; dTE: *p*-value > 0.1; LE: *p*-value > 0.1) groups (Fig. 2i–k, m–o). A significant increase in tumor volume could only be observed in the responder control group at day 10 (*p*-value < 0.05) (Fig. 2h) and in the non-responder treated group at days 7 and 10 (*p*-value < 0.01) (Fig. 2l).

### Early Distinction of Clinical Responders

**Fig. 3.**
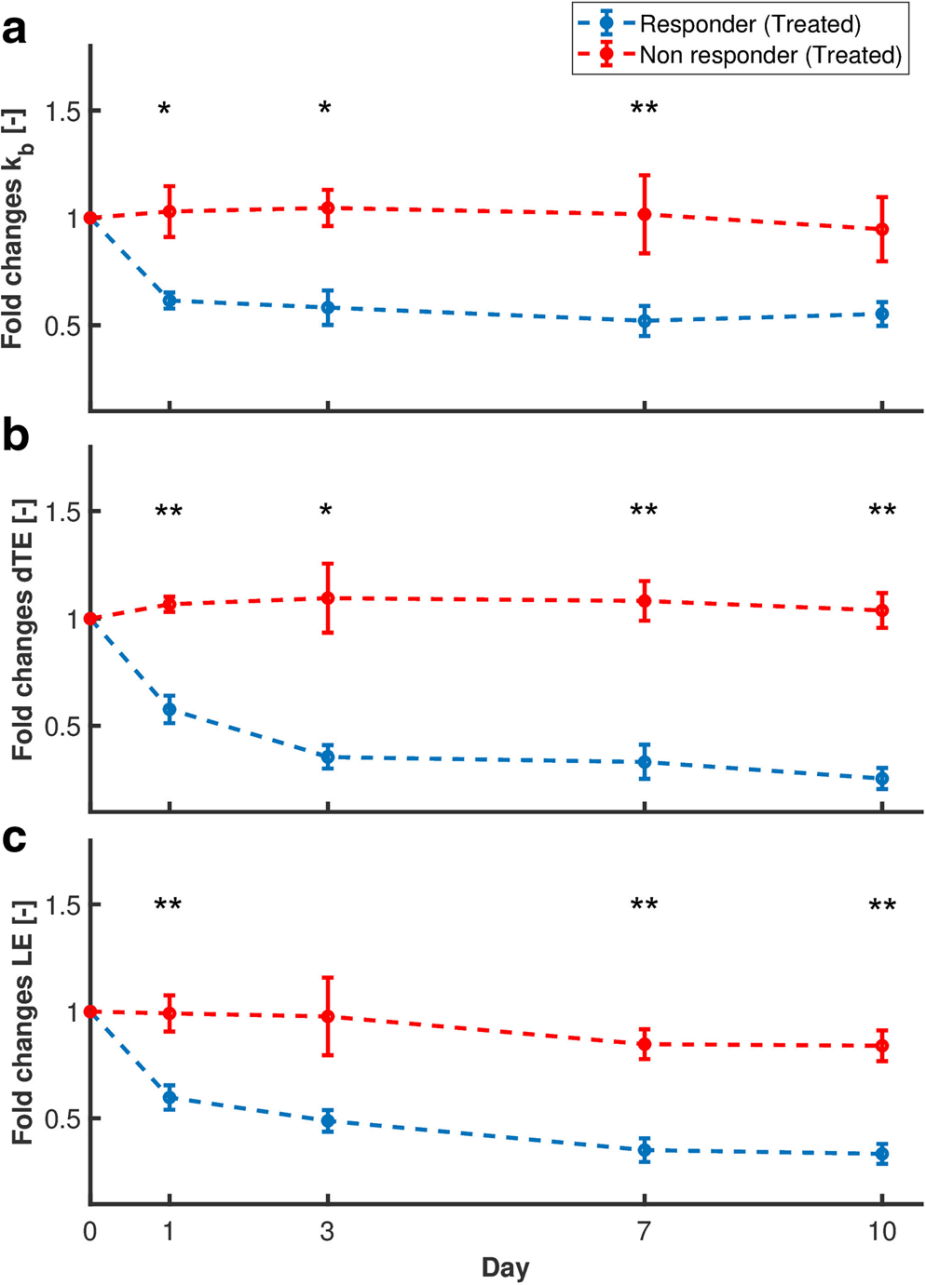
**a**–**c** Fold changes in USMI parameters with respect to day 0 in treated mice, with error bars indicating the standard error of the mean (SEM). Significant differences at each day, tested by the Wilcoxon signed-rank test, are indicated by asterisks (*p-value < 0.05, **p-value < 0.01).

The fold changes in the parameter values in the treated responders and non-responders are compared in Fig. 3. Significant differences were observed already at day 1. Using a threshold of 0.7 change on the pooled values after treatment (including days 1, 3, 7, and 10) provided accuracy in the distinction between responders and non-responders of 85 %, 87.5 %, and 97.5 % for *K*_b_, LE, and dTE, respectively. The results are summarized in Table 1.

**Table 1:** Classification results evaluating the ability of USMI parameters to distinguish between clinical responders and non-responders

Parameter	TP	FP	FN	TN	SENS (%)	SPEC (%)	ACC (%)	PPV (%)	NPV (%)
*k*_b_	16	2	4	18	80.0	90.0	85.0	88.9	81.8
dTE	20	1	0	19	100.0	95.0	97.5	95.2	100.0
LE	19	4	1	16	95.0	80.0	87.5	82.6	94.1

The results are obtained by normalizing each parameter at each day after baseline (days 1, 3, 7, and 10) by the value at baseline (day 0), and then by using a 0.7-fold change as cutoff for positive response to therapy. The results are given in terms of true positives (TP), false positives (FP), false negatives (FN), true negatives (TN), sensitivity (SENS), specificity (SPEC), accuracy (ACC), positive predictive value (PPV), and negative predictive value (NPV)

### Correlation with *Ex-Vivo* Quantification of Angiogenesis

**Fig. 4.**
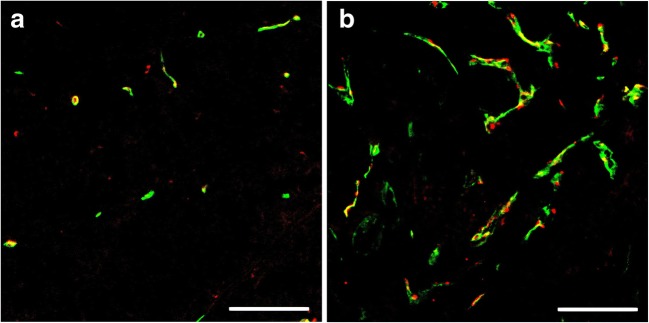
Representative micrographs of tissue slices with merged CD31 staining (green) and VEGFR2 staining (red) in responder tumors, following **a** antiangiogenic treatment and **b** control saline treatment. Scale bar = 100 μm.

**Fig. 5.**
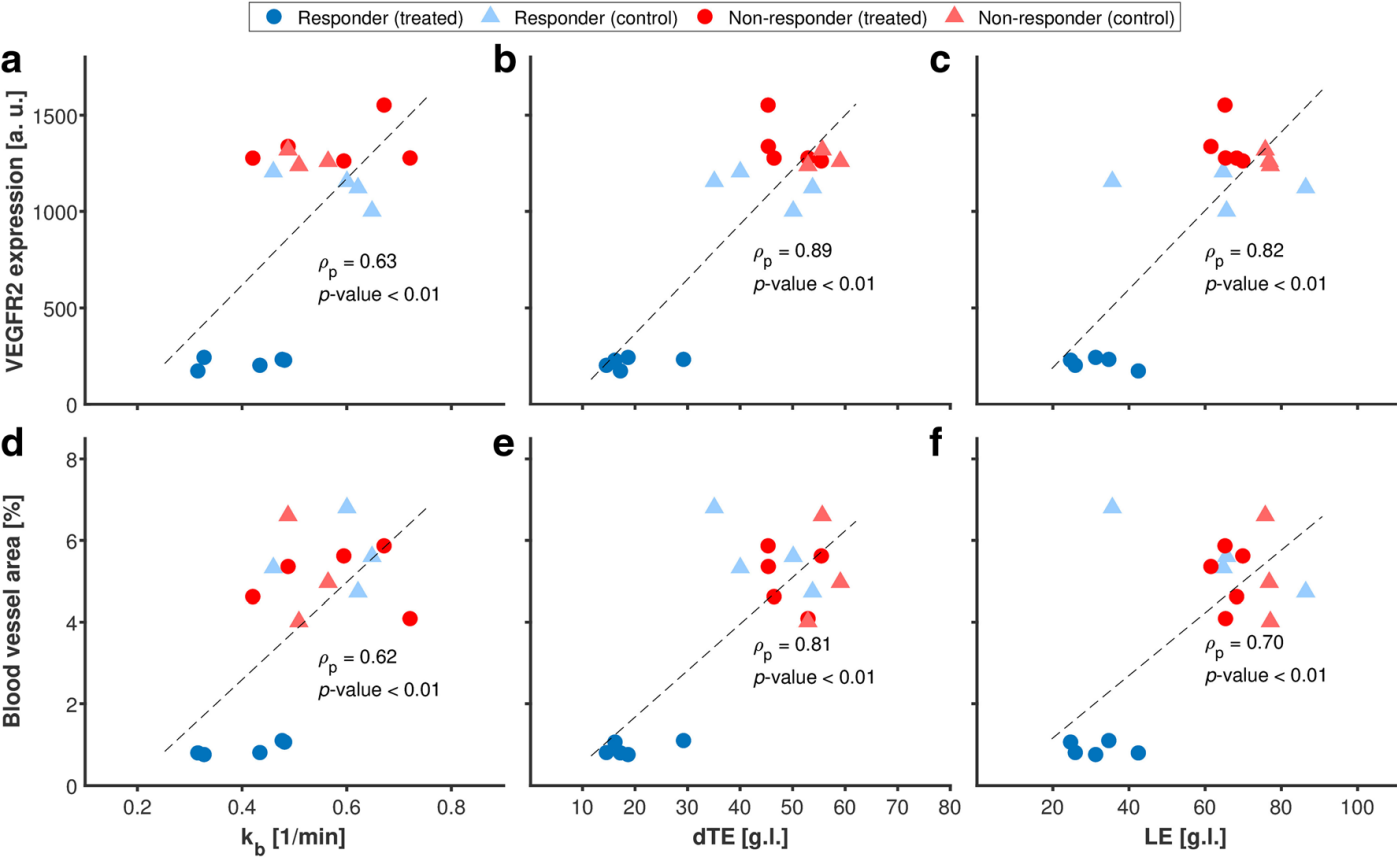
**a**–**f** Scatter plot comparing the *in-vivo* USMI parameter values with the *ex-vivo* immunohistological quantification of VEGFR2 expression levels and the percentage blood vessel area in treated responders (blue circles), control responders (light blue triangles), treated non-responders (red circles), and control non-responders (light red triangles). The regression line (dashed black) is shown, with the corresponding linear correlation coefficient *ρ*_p_ and *p*-value.

Representative histological sections obtained for treated and control responder tumors are shown in Fig. 4. Scatter plots comparing the *in-vivo* USMI parameter values with the *ex-vivo* immunohistological quantification of VEGFR2 expression levels and the percentage blood vessel area are provided in Fig. 5, while the results of the correlation analysis are shown in Table 2. Significant linear and rank correlation was found between all USMI parameters and both VEGFR2 expression and the percentage blood vessel area, except for LE, for which the rank correlation with the percentage blood vessel area was not significant (Spearman *ρ*_s_ = 0.43, *p*-value = 0.09).

**Table 2:** Correlation analysis between the *in-vivo* USMI parameters and the *ex-vivo* immunohistological quantification of VEGFR2 expression levels and the percentage blood vessel area

	VEGFR2 expression		Blood vessel area (%)	
	Person *ρ*_p_ (*p*-value)	Spearman *ρ*_s_ (*p*-value)	Pearson *ρ*_p_ (*p*-value)	Spearman *ρ*_s_ (*p*-value)
*k*_b_	0.63 (< 0.01)	0.50 (< 0.05)	0.62 (< 0.01)	0.62 (< 0.05)
dTE	0.89 (< 0.01)	0.66 (< 0.01)	0.81 (< 0.01)	0.55 (< 0.05)
LE	0.82 (< 0.01)	0.53 (< 0.05)	0.70 (< 0.01)	0.43 (0.09)

### Fitting Performance and Correlation Between USMI Parameters

**Fig. 6.**
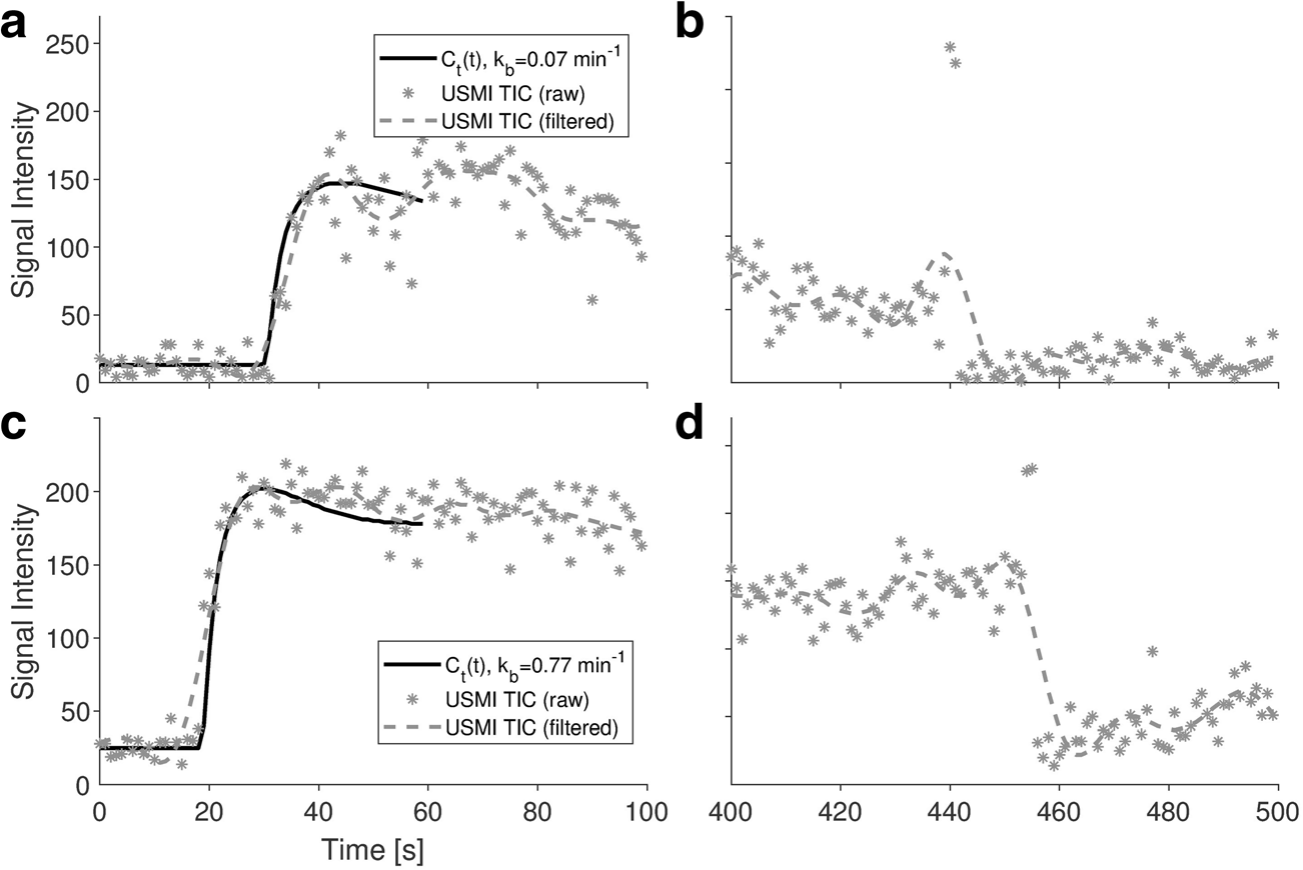
Examples of USMI-derived raw TIC (gray stars), with corresponding filtered TIC (gray dashed line), and FPB fit (black solid line), obtained from two different voxels. **a**, **c** The contrast wash-in (0–100 s) is shown with corresponding model fit. **b**, **d** The late enhancement (400–500 s), including the destructive burst (~ 450 s), is shown. The estimated value of *k*_b_ at each voxel is given in the legend.

An example of USMI-derived TIC with corresponding FPB fit is provided in Fig. 6. The average *R*^2^ of the fit calculated over the whole dataset was 0.88 ± 0.08. Table 3 summarizes the results of the correlation analysis between the USMI parameters. Significant linear and rank correlation was found between all USMI parameters (*p*-value << 0.01). The semi-quantitative parameters showed an expectedly higher correlation between each other (Pearson *ρ*_p_ = 0.88, Spearman *ρ*_s_ = 0.82) than with the quantitative parameter *K*_b_, which showed higher correlation with dTE (Pearson *ρ*_p_ = 0.50, Spearman *ρ*_s_ = 0.46) than with LE (Pearson *ρ*_p_ = 0.46, Spearman *ρ*_s_ = 0.42).

**Table 3:** Correlation analysis between the UMSI parameters, performed by calculating the pairwise linear (Pearson) and rank (Spearman) correlation coefficients

	*k*_b_		dTE		LE	
	Pearson *ρ*_p_ (*p*-value)	Spearman *ρ*_s_ (*p*-value)	Pearson *ρ*_p_ (*p*-value)	Spearman *ρ*_s_ (*p*-value)	Pearson *ρ*_p_ (*p*-value)	Spearman *ρ*_s_ (*p*-value)
*k*_b_	1.00 (<< 0.01)	1.00 (<< 0.01)	0.50 (<< 0.01)	0.49 (<< 0.01)	0.46 (<< 0.01)	0.42 (<< 0.01)
dTE	–	–	1.00 (<< 0.01)	1.00 (<< 0.01)	0.88 (<< 0.01)	0.82 (<< 0.01)
LE	–	–	–	–	1.00 (<< 0.01)	1.00 (<< 0.01)

Corresponding *p*-values are given in parenthesis

## Discussion

In this study, we evaluated semi-quantitative and quantitative USMI for assessment of the response to antiangiogenic treatment in two colon cancer mouse models, simulating clinical responders and non-responders. Our results show the ability of USMI biomarkers to assess the response to treatment earlier than by assessment of tumor volume. A significant decrease in the proposed quantitative parameter *k*_b_ was observed as early as 1 day after treatment, earlier than by all other methods. This suggests USMI to represent a better option for assessment of the early response to therapy than traditional dimension-based criteria, and possibly also suited for assessment of therapies with cytostatic action, whereby no changes in tumor size are expected.

As tumor growth is supported by angiogenesis, stable or increased values of the USMI parameters can be expected in non-responder mice and in responders treated with saline. This is confirmed by our results, showing no significant changes in the USMI parameters, except for the responders treated with bevacizumab. However, non-monotonic variations were observed for the semi-quantitative parameters LE and dTE, suggesting lower reproducibility with respect to quantitative assessment by *k*_b_.

Other imaging modalities are under investigation for monitoring the response to antiangiogenic therapies, including computed tomography (CT) [38], positron emission tomography (PET) [39], and magnetic resonance imaging (MRI) [40]. Although DCE-CT, PET/CT, and MRI have shown to be useful in detection and staging of colorectal cancer and metastases, especially in the preoperative settings [41–43], the use of these modalities for therapy monitoring is hampered by the inherent radiation risk (CT, PET) and by the high cost and low availability (MRI, PET). Combining low cost, widespread availability, portability, and absence of ionizing radiation, USMI is advantageous for therapy monitoring, whereby repeated exams are performed. However, clinical translation of USMI requires more extensive clinical validation, evidencing the need for quantitative biomarkers of therapy response and for a standard clinical protocol to enable reliable evaluation and comparison of findings.

In this study, quantitative analysis was performed by fitting the FPB model to USMI-derived TICs. This model is the solution of a bi-compartmental model describing the concentration of free microbubbles as resulting from a convective dispersion process, and the concentration of bound microbubbles as resulting from a well-mixed compartment, where binding occurs at a rate given by *k*_b_. Since only the first pass of the contrast bolus is considered, the assumption of negligible unbinding can be made, and any effect due to tUCA recirculation can be disregarded. Moreover, fitting the tUCA first pass avoids the need for long acquisition times and for the application of a high-pressure destructive burst, which are instead required by current semi-quantitative methods. Although more complex modeling would permit relaxing these assumptions and fitting the whole curve, the increased number of free parameters may result in inaccurate parameter estimation [3].

*In-vitro* studies have shown very low microbubble detachment also in high-flow and high-shear stress conditions [44], supporting the assumption of negligible unbinding. Moreover, (un)binding kinetics in the order of min^−1^ were reported for the surface size of interest (~ 0.002 mm^2^) [45], confirming the validity of the adiabatic approximation. Regarding the first-pass assumption, although 1-min recirculation time is appropriate in humans, this might not apply to small animals, for which a recirculation time of about 15–20 s can be expected [46, 47]. However, considering that the actual appearance time in the measured TICs was typically larger than 20 s (Fig. 6) and that the tUCA concentration in the second and later bolus passes is typically much lower [35], the first-pass assumption might still represent a valid approximation. A preliminary *in-vivo* validation of the method performed in rats, comparing *k*_b_ estimates obtained for targeted and non-targeted UCA, suggests the model to accurately describe the kinetics of both [48]. However, simulation studies should be performed in the future to evaluate the accuracy, precision, and repeatability of parameter estimation, and its sensitivity to the abovementioned underlying assumptions; also, the signal-to-noise and temporal sampling requirements of the method could be tested *in silico*, possibly proposing an optimized acquisition protocol for improved performance.

Although angiogenesis inhibitors have been used successfully as first- and second-line treatment in some tumor types [7, 8, 49, 50], resistance has been reported in some cases [49, 51, 52]. Early distinction of clinical non-responders is therefore crucial to permit timely adjustments in the therapeutic strategy, possibly improving treatment efficacy, sparing patients the morbidity and severe side effects associated with antiangiogenic therapies [53] and potentially cutting clinical costs due to unnecessary treatment. In our study, comparison of treated responders and non-responders showed USMI parameters to be able to predict the response to therapy already 1 day after treatment initiation. However, only one tumor model responding to treatment and one tumor model showing resistance to the investigated antiangiogenic inhibitor were here compared. Moreover, as the investigated drug and imaging probe targeted the same angiogenic expressions and only short-term monitoring up to 10 days after treatment was here performed, late resistance due to the development of alternative angiogenic pathways could not be investigated [49, 51, 52]. Further preclinical validation, involving different organs, tumor models, and antiangiogenic drugs, and comparison with perfusion assessment and long-term survival criteria are thus necessary to clarify the role of USMI for early prediction of the therapeutic response.

The ability of the proposed *in-vivo* USMI parameters to reflect angiogenesis was validated by comparison with the *ex-vivo* immunohistological assessment of VEGFR2 expression levels and the percentage blood vessel area, performed on excised tumors. Excluding the rank correlation between LE and the percentage blood vessel area (*ρ*_s_ = 0.43, *p*-value = 0.09), significant linear and rank correlation was found between all *in-vivo* and *ex-vivo* angiogenesis biomarkers. In general, higher correlation was found with the VEGFR2 expression levels compared to the percentage blood vessel area. This may be expected considering that the adopted *in-vivo* biomarkers all reflect the binding levels of VEGFR2-targeted microbubbles and thus are more directly related to VEGFR2 expression levels than the percentage blood vessels area, which reflects more structural features of angiogenic vasculature. Moreover, the lower linear correlation found between VEGFR2 expression for *k*_b_, compared to LE and dTE, may be due to the fact that *k*_b_, representing the microbubble binding rate, does not reflect only the degree but also the kinetics of microbubble binding. Further validation in different tumor models may provide greater insight on whether the binding kinetics vary in different tumor types.

Similar conclusions can be drawn from the correlation analysis within the USMI parameters, which showed higher correlation between the semi-quantitative parameters LE and dTE, than between *k*_b_ and LE/dTE. This suggests that *k*_b_ may provide different insights into microbubble binding, while the information given by LE and dTE may be overlapping; this might also explain the surprising result by the FPB-fitting approach, performed only on the first minute and yet providing earlier prediction than late enhancement. In fact, by reflecting the binding kinetics, the quantitative parameter *k*_b_ may represent a more accurate biomarker of angiogenesis as compared to LE and dTE, whose accuracy may be affected by the risk of late bubble detachment and limited by the technical difficulty to obtain a stable concentration plateau before and after the destructive burst. Since *k*_b_ showed lower correlation with LE than dTE, in future work, it might be interesting to investigate whether improved performance can be obtained by the combination of *k*_b_ and LE, which would provide quantitative analysis with an easier and safer acquisition protocol, not requiring the application of a destructive US burst. Information about the LE, for instance, could be exploited for a smarter initialization of *k*_b_, possibly improving estimation convergence and accuracy.

There are some limitations in this study. Although lacking a clear physiological link with the underlying angiogenic processes, other empirical and pharmacokinetic models are available to fit USMI-derived TICs [28–31]. A comparative study should be performed in future work to assess which model and parameters are most suitable for quantitative USMI of cancer angiogenesis. Also, no comparison was here performed with other US methods such as perfusion assessment by conventional contrast-enhanced ultrasound, which has shown promise for early assessment of the response to therapy and good correlation with dimension-based criteria and overall survival [54]. In previous studies, USMI showed ability to detect treatment response much earlier than by assessment of perfusion and tumor volume [15]; moreover, USMI showed higher correlation with VEGFR2 expression levels [14], an established prognostic biomarker of cancer aggressiveness [55–58]. However, since antiangiogenic therapy is mostly used as second-line treatment in conjunction with chemotherapy [6, 8], future research should clarify on the different information provided by molecular and perfusion assessment in relation to the response to combined therapies. Moreover, although the adopted FPB model has shown promise for antiangiogenic therapy monitoring by the quantitative parameter *k*_b_, this study was performed on two mouse xenograft models, for which tumor biology is inherently different than that of humans, and on a limited dataset, with the largest group including five mice. More extensive preclinical validation and feasibility studies in humans are thus necessary to confirm the promising results.

Our findings contribute to the cohort of preclinical studies showing the promise of VEGFR2-targeted microbubbles in the context of angiogenesis imaging and therapy monitoring [14, 19–21, 24]. Currently, no molecularly targeted contrast agent has been approved for clinical use. Initial studies in humans have demonstrated the feasibility and clinical safety of VEGRF2-targeted microbubbles for USMI of prostate [18], breast, and ovarian [19] cancer. However, more extensive validation and the implementation of multi-center studies are required to allow clinical translation. In this context, a standardized quantitative protocol may be useful to improve reproducibility and to facilitate the comparison of findings between different studies and centers, especially important for therapy monitoring, whereby several longitudinal measurements need to be compared. Based on the results of our study, here we suggest the combination of the (semi)quantitative parameters LE and *k*_b_ for a standardized quantification protocol feasible for clinical USMI in the context of antiangiogenic therapy monitoring.
